# Association between Klotho protein and melanoma and nonmelanoma skin cancer in the US adult population: A cross-sectional study

**DOI:** 10.1016/j.jdin.2023.08.014

**Published:** 2023-08-28

**Authors:** Nargiza Sadr, Rehan Qayyum

**Affiliations:** Department of Internal Medicine, Eastern Virginia Medical School, Norfolk, Virginia

**Keywords:** clinical research, dermatology, Klotho protein, medical dermatology, melanoma, nonmelanoma skin cancer, oncology

*To the Editor:* Klotho protein is involved in various physiologic processes, including delayed aging and arteriosclerosis, FGF23 and bone morphogenetic protein signaling, and suppression of oxidative stress.^1^^,^^2^ Animal studies have suggested that Klotho may protect against melanoma and nonmelanoma skin cancer.^3^^,^^4^ However, no large epidemiologic studies have investigated a relationship between Klotho levels and skin cancer risk. Therefore, we used National Health and Nutrition Examination Survey data from 2007-2016 to examine the association between serum α-Klotho protein levels and melanoma and nonmelanoma skin cancer.

Self-report of the skin cancer diagnosis of any type and of melanoma were primary and secondary outcomes, respectively. Klotho levels were measured in individuals aged 40 to 79 years, using the enzyme-linked immunosorbent assay. Participants with information on the outcomes and Klotho levels were included. We conducted survey-weighted logistic regression analysis without and with adjustment for age, gender, race, smoking, alcohol use, obesity (body mass index of >29.9 kg/m^2^), hypertension, diabetes mellitus, the highest level of education attained, and household income to poverty threshold ratio. All analyses were performed using Stata version 16.1 (Stata Corp LP).

Among 13,765 participants, the mean (SD) age was 57.9 (10.9) years, 7098 (51.6%) were women, 5921 (43%) were non-Hispanic White, and 2737 (19.8%) were non-Hispanic Black (Table I). Of the 523 (3.8%) subjects with a history of skin cancer, 110 had melanoma. The mean (SD) Klotho level was 0.85 (0.31) ng/mL. Subjects with a history of skin cancer had significantly lower Klotho levels than those without (0.80 vs 0.86 ng/mL; *P* <.001). In unadjusted linear regression models, each ng/mL increase in Klotho was associated with a 40% lower odds of skin cancer history (odds ratio [OR], 0.60; 95% CI, 0.40-0.90; *P* =.02) (Table II). However, this relationship dissipated in adjusted models. Higher Klotho levels were associated with 37% lower odds of melanoma history, although this relationship did not reach statistical significance in unadjusted and adjusted models, likely because of few subjects with melanoma history (Table II).

**Table I:** Study population characteristics

Variables	Klotho				*P* value
	Quartile 1	Quartile 2	Quartile 3	Quartile 4	
**Age, y**	59.3 (11.1)	58.2 (10.8)	57.6 (10.7)	56.6 (10.6)	.06
**Elderly (>64 y), *N* (%)**	1241 (36.0)	1075 (31.2)	1019 (29.6)	877 (25.5)	<.001
**Females, *N* (%)**	1665 (48.4)	1662 (48.3)	1811 (52.6)	1960 (56.9)	<.001
**Race, *N* (%)**					
African Americans	698 (20.3)	569 (16.5)	585 (17.0)	875 (25.4)	<.001
Caucasians	1555 (45.2)	1592 (46.2)	1504 (43.7)	1270 (36.9)	
Other	1189 (34.5)	1281 (37.2)	1351 (39.3)	1296 (37.7)	
**Smoking status, *N* (%)**					
Nonsmokers	1575 (22.7)	1703 (24.1)	1829 (25.8)	1968 (27.8)	<.001
Past smokers	1091 (27.4)	1036 (26.0)	975 (24.5)	884 (22.2)	
Current smokers	771 (28.6)	702 (26.0)	635 (23.5)	588 (21.8)	
**Alcohol use, *N* (%)**					
Nondrinkers	631 (22.2)	682 (24.0)	700 (24.7)	825 (29.1)	<.001
Light drinkers	2264 (25.8)	2203 (25.1)	2200 (25.0)	2119 (24.1)	
Heavy drinkers	334 (29.2)	312 (27.3)	276 (24.1)	222 (19.4)	
**Highest education level**					
Less than high school	1010 (29.38)	968 (28.13)	951 (27.67)	961 (27.93)	<.001
High school	827 (24.05)	747 (21.71)	771 (22.43)	704 (20.46)	
Some college	917 (26.67)	928 (26.97)	939 (27.32)	929 (27.0)	
College graduate	684 (19.9)	798 (23.19)	776 (22.58)	847 (24.61)	
**Household income to poverty threshold ratio**					
0-1	653 (20.82)	616 (19.46)	622 (19.52)	652 (20.75)	.54
>1 to 2	855 (27.26)	830 (26.22)	817 (25.64)	805 (25.62)	
>2 to 3	471 (15.02)	460 (14.53)	480 (15.07)	452 (14.39)	
>3 to 4	324 (10.33)	343 (10.84)	339 (10.64)	346 (11.01)	
>4	833 (26.56)	916 (28.94)	928 (29.13)	887 (28.23)	
**Melanoma history, *N* (%)**	33 (0.96)	33 (0.96)	28 (0.81)	16 (0.46)	.07
**Skin cancer history, *N* (%)**	149 (4.33)	137 (3.98)	143 (4.16)	94 (2.73)	.002
**Hypertension, *N* (%)**	1886 (54.8)	1735 (50.4)	1647 (47.9)	1678 (48.8)	<.001
**Diabetes mellitus, *N* (%)**	689 (20.0)	602 (17.5)	599 (17.4)	705 (20.5)	<.001
**Obesity, *N* (%)**	1451 (42.8)	1411 (41.6)	1410 (41.5)	1411 (41.4)	.61
**Body mass index, kg/m**^**2**^	29.9 (6.4)	29.7 (6.6)	29.6 (6.7)	29.7 (7.0)	.49
**Klotho, ng/mL**	0.54 (0.08)	0.73 (0.04)	0.89 (0.05)	1.3 (0.30)	<.001

**Table II:** Odds of history of any skin cancer or melanoma with serum Klotho protein levels

Variable	History of any skin cancer∗	History of melanoma∗
Continuous Klotho, ng/mL		
Unadjusted	0.60 (0.40-0.90); 0.02	0.63 (0.25-1.62); 0.33
Adjusted	0.80 (0.54-1.20); 0.29	0.7 (0.28-1.73); 0.43
Klotho quartiles		
Unadjusted Q1	REF	REF
Q2	0.87 (0.64-1.18); 0.37	1.03 (0.58 to 1.83); 0.91
Q3	1.08 (0.82-1.42); 0.57	0.92 (0.56 to 1.52); 0.75
Q4	0.66 (0.45-0.96); 0.03	0.69 (0.36 to 1.33); 0.26
Adjusted Q1	REF	REF
Q2	0.92 (0.66-1.27); 0.60	0.99 (0.56-1.75); 0.97
Q3	1.25 (0.92-1.68); 0.15	1.13 (0.69-1.86); 0.61
Q4	0.82 (0.55-1.23); 0.33	0.73 (0.34-1.57); 0.41

∗ Results are presented as odds ratio (95% CI); *P* value. Adjusted models also included age, gender, race, obesity, alcohol use, smoking, hypertension, diabetes mellitus, highest level of education attained, and household income to poverty threshold ratio.

The highest quartile of Klotho had significant 34% lower odds of skin cancer (OR, 0.66; 95% CI, 0.45-0.96; *P* =.03) than the lowest quartile; the odds for melanoma were not significant (OR, 0.69; 95%CI, 0.36-1.33; *P* =.26). After adjustment for potential confounders, the relationship between Klotho quartiles and nonmelanoma and melanoma skin cancer was no longer significant (Table II).

We found a significant inverse relationship between Klotho levels and a history of skin cancer. However, this association disappeared after accounting for potential confounders; possibly a confounder was an important mediator. The study examines the relationship between relatively stable and not transient variations in Klotho levels and distant cancer. Klotho levels are partly under genetic control, and therefore, relatively stable.^5^ Although nonmelanoma skin cancers are a heterogeneous group, Klotho proteins decrease the risk of a variety of cancers likely through shared malignant transformation pathways, allowing us to cluster nonmelanoma skin cancers under 1 category. Although causality cannot be inferred because of cross-sectional design, to our knowledge, it is the first study examining the relationship. Further research is needed to clarify the relationship between Klotho and skin cancer.

## Conflicts of interest

None disclosed.
